# Unsupervised detection of regulatory gene expression information in different genomic regions enables gene expression ranking

**DOI:** 10.1186/s12859-017-1497-z

**Published:** 2017-02-01

**Authors:** Zohar Zafrir, Tamir Tuller

**Affiliations:** 1 Department of Biomedical Engineering, Tel Aviv University, P.O. Box 39040, Tel Aviv, 6997801 Israel; 2 Sagol School of Neuroscience, Tel Aviv University, P.O. Box 39040, Tel Aviv, 6997801 Israel

**Keywords:** Gene expression, Intron evolution, Transcript evolution

## Abstract

**Background:**

The regulation of all gene expression steps (e.g., Transcription, RNA processing, Translation, and mRNA Degradation) is known to be primarily encoded in different parts of genes and in genomic regions in proximity to genes (e.g., promoters, untranslated regions, coding regions, introns, etc.). However, the entire gene expression codes and the genomic regions where they are encoded are still unknown.

**Results:**

Here, we employ an unsupervised approach to estimate the concentration of gene expression codes in different non-coding parts of genes and transcripts, such as introns and untranslated regions, focusing on three model organisms (*Escherichia coli*, *Saccharomyces cerevisiae*, and *Schizosaccharomyces pombe*). Our analyses support the conjecture that regions adjacent to the beginning and end of ORFs and the beginning and end of introns tend to include higher concentration of gene expression information relatively to regions further away. In addition, we report the exact regions with elevated concentration of gene expression codes. Furthermore, we demonstrate that the concentration of these codes in different genetic regions is correlated with the expression levels of the corresponding genes, and with splicing efficiency measurements and meiotic stage gene expression measurements in *S. cerevisiae*.

**Conclusion:**

We suggest that these discoveries improve our understanding of gene expression regulation and evolution; they can also be used for developing improved models of genome/gene evolution and for engineering gene expression in various biotechnological and synthetic biology applications.

**Electronic supplementary material:**

The online version of this article (doi:10.1186/s12859-017-1497-z) contains supplementary material, which is available to authorized users.

## Background

Gene expression codes are known to be partially encoded in various genomic regions [1–6] and are related to all gene expression steps (e.g., Transcription, RNA processing, Translation, Post-translation modifications, and Degradation). These codes are encoded in different parts of the genome such as promoters, untranslated regions (UTRs), coding sequence (CDS) regions, introns, etc. However, the relevant codes and exact genomic regions where gene expression is encoded are still partially unknown, specifically in organisms that are not widely studied. For instance: the methanogenic archaeon *Methanopyrus kandleri* which is living in extreme heat and pressure conditions [7, 8], *Ciona intestinalis* - a sea squirt living in shallow ocean water [9, 10], *Mycoplasma penetrans* - a species of *Mycoplasmataceae* that infects humans in the urogenital and respiratory tracts [11, 12], the human fungal pathogen *Cryptococcus neoformans* [13, 14], and *Rhodotorula* sp. JG1b – a eurypsychrophilic yeast that was recently sequenced in Antarctica [15]).

The conventional approaches for deciphering and understanding gene expression codes and ranking genetic elements (such as promoters, introns, etc.) are based on evaluating their effect via various types of large scale measurements: mRNA levels [16–18], protein abundance (PA) [19], ribosome densities (RDs) [20], transcription factors (TFs) binding sites [21], methylation levels [22], three dimensional genomic conformation [23, 24], and more. These approaches have proven to be useful in many contexts. However, their major limitation is the fact that they are all based on comprehensive large scale gene expression measurements; such high quality data exist in present for only a few dozen organisms, while today there are thousands of organisms with sequenced genomes.

**Fig. 1:**
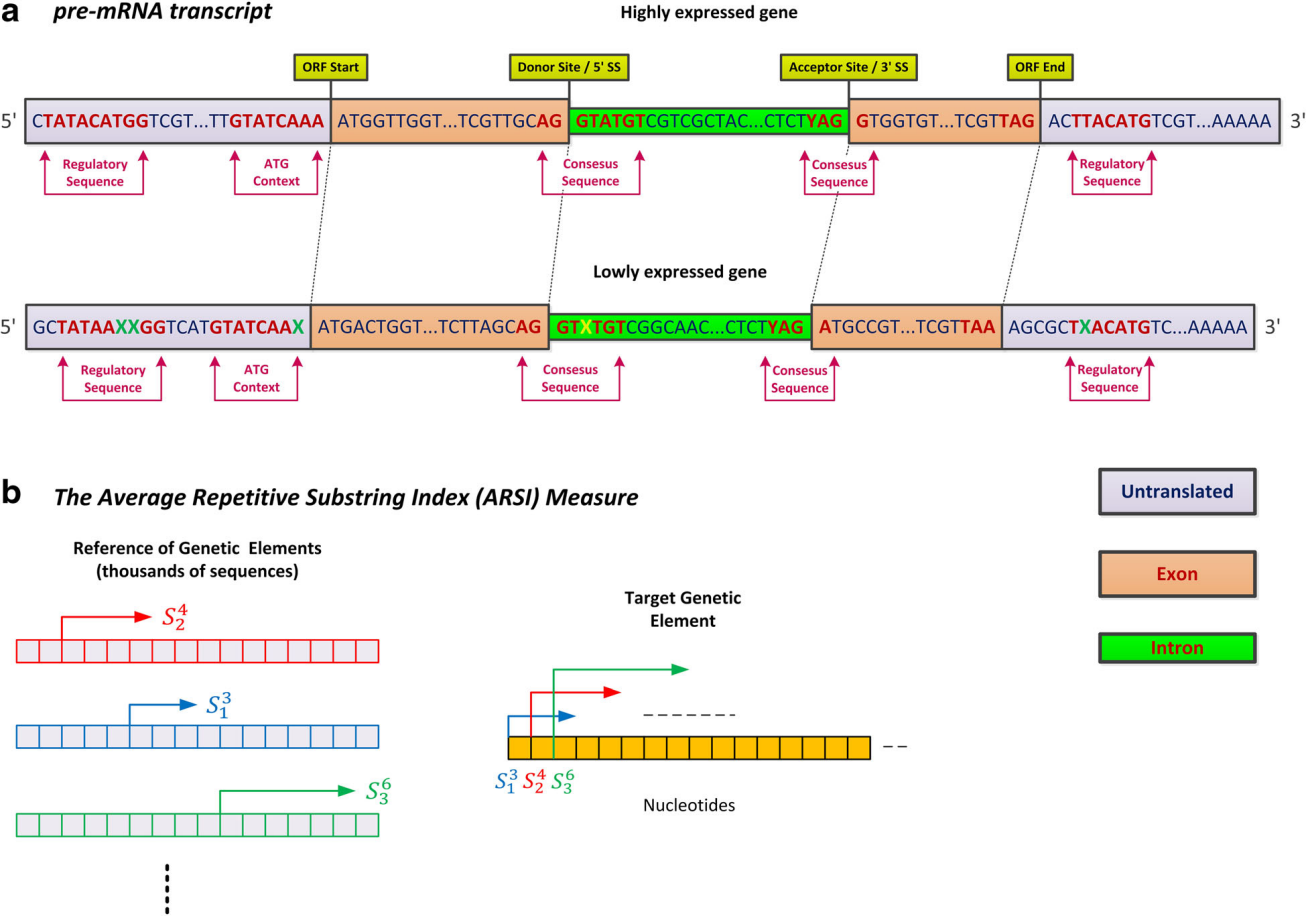
Illustration of the various genomic regions containing interleaved regulatory motif sequences and an illustration of the Average Repetitive Substring Index (*ARSI*) measure. **a** The pre-mRNA transcript contains different sections comprising of interleaved regulatory sequence motifs, which affect gene expression; these regions include exons, introns, and untranslated regions (UTRs). Transcripts of highly expressed genes tend to contain motifs with the precise sequences. However, in transcripts originating from lowly expressed genes these motifs are more likely to acquire mutations, affecting their gene expression; this leads to a lower *ARSI* score for these genes. **b** In order to compute the *ARSI* measure for a certain sequence we find for each nucleotide position in the sequence the longest substring that starts in this position and appears in one of the reference set of genetic sequence elements. The score is based on the average over the lengths of all these substrings; see more details in the Methods section and in [25]

A possible solution to these limitations was recently presented by [25] for studying and engineering coding regions (i.e. open reading frames; ORFs). The Average Repetitive Substring Index, or *ARSI*, is an unsupervised approach for exploiting unexplored high dimensional information and codes related to the way gene expression is encoded in the ORF. This method, based solely on the genomic sequence of the analyzed organism, computes the tendency of each coding region (or any other genetic element for that matter) to include long substrings that appear in other CDSs of the organism [25]. It is based on the assumption that evolution shapes CDSs such that they include various motifs (up to few dozen nucleotides in length), which are related to various gene expression regulatory steps. Since highly expressed genes undergo evolution to include optimal versions of these motifs they are expected to share sub-sequences/motifs with other genes (e.g., other highly expressed genes), resulting with higher *ARSI* score. On the other hand, lowly expressed genes are expected to have less optimized motifs, i.e. versions of the optimal motifs with various random ‘mutations’; these mutations ‘break’ these sequence motifs and result in lower *ARSI* score; see Fig. 1a. The *ARSI* score for a given sequence is determined by finding for each nucleotide position in that sequence the longest substring that also appears in (at least) one genetic element sequence of a reference set of sequences. For example, the reference set can include (or be related to) the highly expressed genes or all the genes in a given organism; see Methods and Fig. 1b.

In this study we expand our scope and generalized it to study the suspected regulatory information found in various regions of the DNA and pre-mRNA transcripts, in a single nucleotide resolution; this includes non-coding regions both in eukaryotes and prokaryotes. Specifically, we focused on introns, exons, UTRs, and on the boundary regions between them, i.e. the exon-intron, the 5’UTR-ORF, and the ORF-3’UTR junctions. Among others, we demonstrate how this universal approach can be used for (1) ranking genomic elements according to their optimality in terms of gene expression regulation and (2) detecting regions that are relatively highly populated with many gene expression codes; our analysis performed comparisons to randomized (Null) models that preserve basic properties of the original sequence.

## Methods

### The analyzed organisms

The bacteria *E. coli* is one of the most well studied model organism and was chosen as a representative of prokaryotes [26]. The two fungi analyzed here (*S. cerevisiae* and *S. pombe*) were chosen since they are well-studied organisms, which have diverged more than 400 million years ago [27]. These organisms have fully sequenced genomes, and their exons and introns are very well annotated.

### Sequence and gene expression information

The ORFs and intron-containing genes sequence information for *S. cerevisiae* (strain 288C) was taken from SGD [28] and the Ares lab database [29]. *S. pombe* genome information was taken from the PomBase database (Assembly 16) [30] and the original full genome sequencing obtained by [31]. The genome of *E. coli* (K-12, MG1655) was downloaded from the NCBI website (https://www.ncbi.nlm.nih.gov/). The protein abundance (PA) information for all organisms was taken from PaxDb [19]. The mRNA levels for *S. cerevisiae* were obtained by integration of the following data sets: [20, 32, 33]. Levels of mRNA for *E. coli* were taken from [34]. The mRNA levels for *S. pombe* are based on [35]. See full details in [36].

### Computing the ARSI score

The *ARSI* score was determined based to the following scheme: A given gene, transcript, or genetic region (UTR, intron, CDS, etc.) *P*, can be described as a sequence of nucleotides *S*; thus, the measure is based on the tendency of substrings in *S* to appear in other genetic elements, i.e. in a reference set *G*. Hence, computing the *ARSI (G,S)* score of a specified sequence *(S)* given a reference set of genomic elements *(G)* is done in two steps (see Fig. 1b): 1) For each position *i* in the sequence *S* find the longest substring *S*_*i*_^*j*^ that starts in that position and appears in at least one of the sequences of the reference set *G*. 2) Let |*S*| denote the length of a sequence *S*; the *ARSI* of *S* is the mean length of all the substrings *S*_*i*_^*j*^, i.e. *ARSI* = ∑|*s*_*i*_^*j*^|/|*S*|.

Please note that the *ARSI* measure is based on a reference genome of a given organism, and therefore is not expected to be affected by various sequencing errors/biases that appear in Next Generation Sequencing (NGS) experiments. Specifically, in this study the error rate is very low for the analyzed organisms (less than 1 to 1000). As these errors distribute relatively uniformly, their effect the *ARSI* score is negligible: for example in *E. coli* the Spearman correlation between the *ARSI* scores and the one obtains for a simulation with uniform error rate of 1:1000 is higher than 0.99 (*p* < 5 · 10^−323^) for all 100 such randomization that were performed.

### Computing the ARSI profiles

The *ARSI* profiles were computed as follows: we used various sliding window sizes (*WL* equals to 31, 41, 51, and 71 nucleotides) focusing on the region of interest (5’UTR/ORF/Intron/3’UTR) and its flanking sequences; for every region we computed the *ARSI* score for all sliding windows, with a single nucleotide shift. Let *ARSI*_*WL*(*i*) denote the score of a window size of *WL* nucleotides, centered on the *i*_*th*_ nucleotide of the gene’s pre-mRNA transcript. The profile of gene *j* was defined as the vector of the *ARSI* values assigned to *n* sliding windows of size *WL*, i.e. $ARSI\_ W{L}_{Gen{ e}_j}=\left( ARSI\_ W{L}^j(1), ARSI\_ W{L}^j(2),\dots, ARSI\_ W{L}^j(n)\right)$. All genes were aligned according to their relevant location (ORF start, 5’SS, 3’SS, and ORF end). Let *i*_*loc*_ denote the positions of the region of interest. The profiles of mean *ARSI* were calculated as:

$\overline{ARS{I_{WL}}_{loc}}=\left(\overline{ARS I\left({i}_{loc}-\left( n-\frac{1}{2}\right)\cdot WL+1\right)},\dots, \overline{ARS I\left({i}_{loc}\right)},\dots, \overline{ARS I\left({i}_{loc}+\left( n-\frac{1}{2}\right) \cdot WL\right)}\right)$, where $\overline{ARSI\_ WL(i)}$ is the average *ARSI* in position *i* when considering all genes long enough to have a value in this position, and (*n* − 1/2) ⋅ *WL* is the number of nucleotides in the complete analyzed exonic and intronic regions (we used *n* = 4); see illustrated in Additional file 1: Figure S3. For calculation simplicity, genes containing *m* > 1 introns were duplicated *m* times. Thus, for each duplicate, a different intron was retained while the other introns were extracted.

### Generating the null models

The randomized models were designed to conserve the encoded protein information and intronic and UTR properties, by maintaining codon-usage bias (CUB), canonical splicing signals, and GC content. Introns nucleotides were uniformly permutated, per gene, maintaining intronic consensus sequences (5’SS, BS, and 3’SS) and GC content. Untranslated regions such as 5’UTR and 3’UTR were also randomized, using a cyclic shift of the nucleotides that maintained their GC content properties; 5’UTR ATG context was also maintained. We used three basic randomization schemes to generate the random sets: (*a*) Codons only, (*b*) Introns only, and (*c*) UTRs only. A combination of these schemes was later applied, i.e. (*a*) + (*b*) + (*c*), and is used throughout the study. See full details in [37]. An illustrated of the randomization models can be seen in Additional file 1: Figure S4.

### Computing Z-scores based on the null models

Z-score (or standard normal distribution scoring) is a statistical measure, which can be used for quantitative selection level evaluation; this is done by a comparison of the real signal to a randomized one. Hence, higher Z-score value is related to higher p-value, corresponding to the rejection of our null model (which is described in the previous sub-section; see also [37]).

### Partial correlation analysis

Partial correlation analysis is aimed at finding the correlation between two variables after removing the effects of other variables; the partial correlation coefficient *ρ*_*xy*,*z*_between *X* and *Y* given a set of *n* controlling variables *Z* = {*Z*1, *Z*2 …, *Zn*} is the correlation between the residuals *R*_*X*_ and *R*_*Y*_ resulting from the linear regression of *X* with *Z* and of *Y* with *Z*, respectively; the approach can be generalized to deal with Spearman correlation [38].

### Synthetic YiFP reporter library building and analysis

The building of the synthetic reporter library facilitating the assessment of native budding yeast introns embedded in a Yellow Fluorescent Protein (YFP), was previously reported [39–41]. The system contains 240 strains (termed YiFP) and allows dynamic measurements of their relative YFP expression levels, which is related to intronic splicing efficiency in *S. cerevisiae*; see full details in [41].

### Analysis of mRNA-seq and Ribo-seq measurements

The ribosomal profiling (or Ribo-seq) is a method that gives quantitative information of ribosome footprints in a single nucleotide resolution [20]. Ribo-seq/mRNA-seq raw data was obtained the from the NCBI GEO database [16] (accession GSE34082). Transcript sequences were obtained from EnsEMBL for *S. cerevisiae* (R64-1-1, Ensembl release 78). We trimmed 3’poly-A adaptors from the reads using Cutadapt, version 1.8.3 [42]. Following, we utilized Bowtie [43] to map them to the *S. cerevisiae* transcriptome (version 1.1.1). In the first phase (for Ribo-seq reads only), we discarded reads that mapped to rRNA and tRNA sequences (Bowtie parameters ‘–n 2 –seedlen 23 –k 1 --norc’). In the second phase (for both Ribo-seq and mRNA-seq reads), we mapped the remaining reads to the transcriptome (Bowtie parameters ‘–v 2 –a --strata --best --norc –m 200’). We tried to extend alignments to their maximal length by comparing the poly-A adaptor with the aligned transcript until reaching the maximal allowed error (i.e. two mismatches across the read, with 3'end mismatches avoided). We filtered out reads longer than 32 nt or shorter than 23 nt for Ribo-seq reads, and filtered out reads longer than 40 nt or shorter than 25 nt for mRNA-seq reads. Unique alignments were first assigned to the RNA/ribosome occupancy profiles. For multiple alignments, the best alignments in terms of number of mismatches were kept. Then, multiple aligned reads were distributed between locations according to the distribution of unique ribosomal/RNA reads in the respective surrounding regions. To this end, a 100 nt window was used to compute the read count density *RCD*_*i*_ (total read counts in the window divided by length, based on unique reads) in vicinity of the *M* multiple aligned positions in the transcriptome, and the fraction of a read assigned to each position was determined as: $R C{D}_i/{\displaystyle \sum_{j=1}^M} R C{D}_j$. For ribosome footprints, the location of the A-site was set 15 nt downstream of the 5' of the read.

## Results

During gene expression steps the genetic material (DNA, pre-mRNA, and mature mRNA) interacts with many intracellular molecules and complexes such as the polymerase [1], the spliceosome [36, 44, 45], pre-initiation complexes [46, 47], ribosomes [48], tRNAs, miRNAs, and various proteins and factors [5, 49, 50]; see illustration in Additional file 1: Figure S1. The affinity of these interactions is affected by the nucleotide composition in various parts of the gene, transcript, and in proximity to genes [1–5, 21, 46, 49, 51–56]. Hence, we aimed at estimating the concentration of gene expression codes in different coding and ***non-coding*** parts of genes and transcripts such as exons, introns, and UTRs using the *ARSI* measure. In addition, we aimed at quantifying the relation between the estimation of these code concentration and gene expression; see Methods and Fig. 1. To this end we analyzed the genome of one prokaryote (*Escherichia. coli*) and two eukaryotes (the fungi *Saccharomyces. cerevisiae* and *Schizosaccharomyces. pombe*; for further details regarding these organisms see the Methods section).

### Evidence that high dimensional gene expression codes appear in various transcript regions

First, we analyzed the pre-mRNA transcript, dividing it into separate regions: 5’UTRs, ORFs, introns, 3’UTRs, and the 250 nt flaking upstream and downstream sequences from the 5’UTR start and the 3’UTR end, respectively. Specifically, we considered all the genetic elements in the organismal genome related to each region as the reference genome, excluding the current one. First, we computed the *ARSI* measure for the real and randomized models; the randomized versions preserve some of the original sequence properties (e.g., GC content in non-coding regions and codon distribution and the encoded proteins related to coding regions); however, they do not include the same higher dimensional distributions (see details in Methods). For each genetic region, we calculated its *ARSI* score, which is the mean over the maximum substring length of each of its nucleotide positions that can be found in all the other genetic regions. For *E. coli* this was done using 4136 genes with measured protein levels [19]. For *S. cerevisiae* we used 3,804 genes that have observed protein levels [57] and 279 intron-containing genes [28, 58]. For *S. pombe* we used 5012 genes with measured protein levels [19] and 2337 intron-containing genes with a total of 4747 introns [30, 31].

**Fig. 2:**
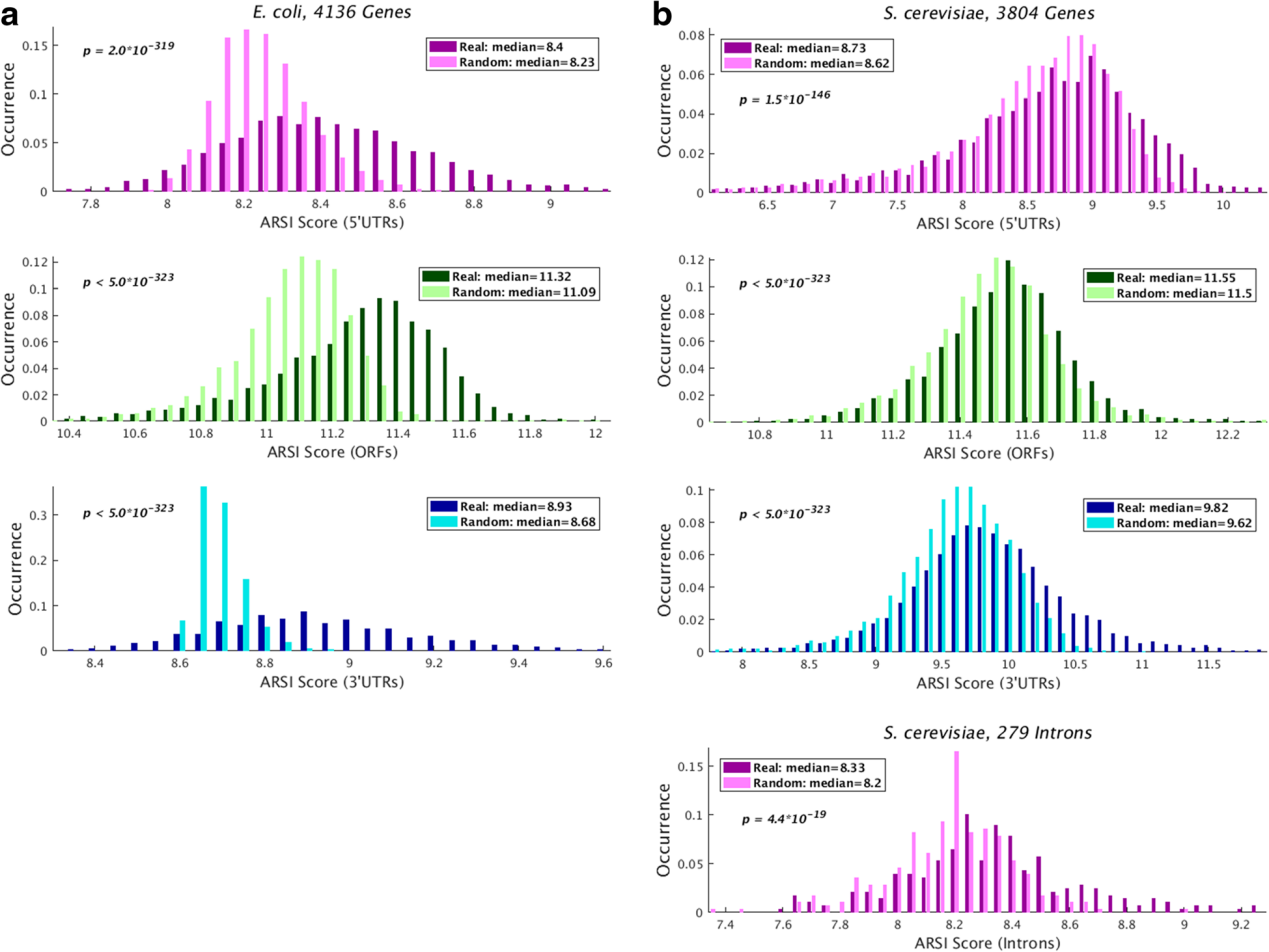
*ARSI* score distribution for the real and randomized models in various transcript regions for *E. coli* (**a**) and *S. cerevisiae* (**b**). **a** The *ARSI* values for *E. coli* in the real transcriptome are significantly higher than the randomized models in all the examined regions (5’UTR/ORF/3’UTR, top/middle/bottom, respectively; *p* < 2 · 10^−319^, Wilcoxon signed-rank test that is a paired test). **b** The *ARSI* values for *S. cerevisiae* in the real transcriptome are significantly higher than the randomized models in all the examined regions (5'UTR/ORF/3'UTR/Intron; *p* < 4.4 · 10^−19^, Wilcoxon signed-rank test). These results indicate that the real sequences tend to include longer substrings in comparison to the randomized ones

The summary of the *ARSI* scores distribution in the real vs. the randomized genome appears in Fig. 2. As can be seen, the real sequence elements in *E. coli* contain significantly more encoded information than the randomized ones (e.g., median score of 8.4 vs. 8.23 in the 5’UTR sequences, respectively; *p* = 2 · 10^−319^, Wilcoxon signed-rank test that is a paired test). Similar results were observed in *S. cerevisiae* (e.g., median score of 8.33 vs. 8.2 in the intron sequences, respectively; *p* < 4.4 · 10^−19^) and *S. pombe* (see Additional file 1: Figure S2a). It is important to emphasize that a small change in the *ARSI* score may be very significant in its effect on the expression levels and ranking of genes, since regulatory high dimensional motifs are expected to appear in relatively small fraction of the genetic material; see Additional file 1: Figure S1.

### Detection of the regions in the DNA with high concentration of gene expression regulatory information

**Fig. 3:**
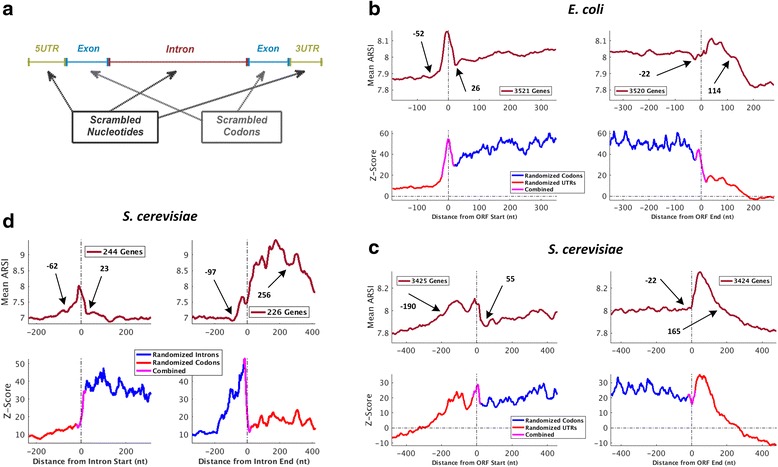
Information concentration (mean *ARSI; top*) and selection (Z-score; bottom) profiles in various transcript regions for *E. coli* and *S. cerevisiae*. The sequences are aligned to the ORF’S start, 5'SS, 3'SS, and ORF end; in *E. coli*, which is prokaryote, only the ORF alignments were generated. **a** The profiles correspond to the *ARSI* score of the actual genomic regions in comparison to the randomized ones, using several sequence randomization models of the actual transcriptome that maintain consensus sequences and control for codon-usage bias (CUB) and GC content in various regions (including coding region, intron, and UTR randomizations; see also details in Methods and Additional file 1: Figures S3, S4): the randomized codon model includes scrambled exonic sequences; the randomized intron model includes scrambled intronic sequences; the randomized UTR models include scrambled untranslated sequences. **b** *E. coli* profiles for the mature mRNA. **c** *S. cerevisiae* profiles for the mature mRNA. **d** *S. cerevisiae* profiles for the pre-mRNA. The profiles show that more information is found in the ORF start, rather than downstream in the ORF; around the intronic splice sites the signal is stronger, as well as downstream from the ORF’s end. In addition, the selective pressure on the transcript sequence is stronger in these locations. This suggests the possible enrichment of regulatory sequence motifs in these regions; the distance from the ORF/5’SS/3’SS is relative to the center of the sliding window; sliding window size is 41 nt; other window sizes showed similar results

Following, we focused on the coding sequence and exon-intron boundaries, (i.e. the regions surrounding the start codon, the stop codon, and the donor and acceptor splice sites), aimed to systematically infer regions that are in preference for higher concentration of regulatory information, at a single nucleotide resolution. To this end, we used a sliding window scheme with varying window sizes of 31–71 nt. For each window, we computed the local *ARSI* score for all genomic elements, to build an averaged profile; see Methods and Additional file 1: Figure S3. Next, and in order to provide evidence of selection and estimate the level of condition-specific expression, we used local Z-score profiles: these profiles include deviation of the actual *ARSI* score from what is expected by the randomized/Null models in standard-deviation units (see Fig. 3a, Additional file 1: Figure S4, and Methods); thus, higher Z-score is related to higher p-value, corresponding to the rejection of our null model.

Figure 3b–d shows the mean assembled profiles over the analyzed genomic regions, aligned to the ORF’s start and end (b, c, left and right, respectively), 5’SS (d, left), and 3’SS (d, right), and using a sliding window size of 41 nt. As can be seen, for the analyzed organisms, there is a clear ascent in the *ARSI* score near the regional boundaries. In *E. coli* there is a noticeable peak surrounding the start codon (nucleotides −52 to 26, relative to the ORF’s start) with a corresponding Z-score of up to 54.5. Similarly, in *S. cerevisiae* there is a noticeable peak following the annotated stop codon (nucleotides −22 to 165, relative to the ORF’s end). When looking on *S. cerevisiae* pre-mRNA of intron-containing genes aligned to the 3’SS, we can see a region with increased gene expression code concentration extending from 97 nt upstream from the acceptor site to 256 nt inside the downstream exons. Results for *S. pombe* can be seen in Additional file 1: Figure S2b, c. It is known that the splice sites and ORF end are populated with many regulatory signals [1, 3, 5, 6, 36, 37, 56]; Thus, these finding demonstrate how the *ARSI* can be used for detecting region with regulatory information.

### High correlation between the ARSI score of various genetic elements and the expression levels of the corresponding genes

Next, we aimed at checking the relation between the *ARSI* scores in the aforementioned regions and expression levels, aiming to show that the *ARSI* score tends to be higher for highly expressed genes. We indeed found significant correlation with all *E. coli* and in *S. cerevisiae* genes, respectively. In addition, the correlation was very high for intron-containing genes in *S. cerevisiae* (*r* = 0.55, *p* = 7.3 · 10^−23^; Spearman correlation of the ORF sequences with mRNA levels), which are known to be very highly expressed. Interestingly, this is was also true when considering 240 synthetic YiFP library genes in *S. cerevisiae* (*r* = 0.27, *p* = 7.2 · 10^−5^) taken from [41]. Correlation remains significant even while controlling for the sequence length (using partial correlation; see Methods). See full details in Additional file 2: Table S1.

Following, and in on order to understand if the *ARSI* can rank genes based on inspecting their condition-specific gene expression, we analyzed mRNA-seq and ribosomal profiling (or Ribo-seq; see [20]) measurements of meiotic cell cycle stages in *S. cerevisiae* taken from [59]. Specifically, we ranked the genes based on their RD and mRNA levels for various genomic regions (i.e. 5’UTR, ORF, and 3’UTR). We than analyzed the association of *ARSI* scores with these measurements, per stage (see details in the Methods). We found that the correlation between the *ARSI* score and the mRNA-seq/Ribo-seq data varies along the cell cycle with a correlation of up to 0.31/0.35 (see Additional file 1: Figure S5; *p* < 1.6 · 10^−6^ and *p* < 3 · 10^−2^, respectively). While the significant time point with the highest RNA-seq correlation is related to the spores ‘stage’, the correlation usually seems relatively similar across the different conditions. This may suggests that, at least in this example, the gene expression information detected by the *ARSI* corresponds in a relatively uniform manner (in terms of the expression levels of genes and positions within genes) to different meiotic cell cycle stages. This makes sense since we expect all cellular conditions (e.g., cell cycle stages) to constraint the evolution of transcripts and that the *ARSI* measure is aimed to capture all relevant gene expression signals. Detailed correlation information can be found in Additional file 2: Table S2.

**Fig. 4:**
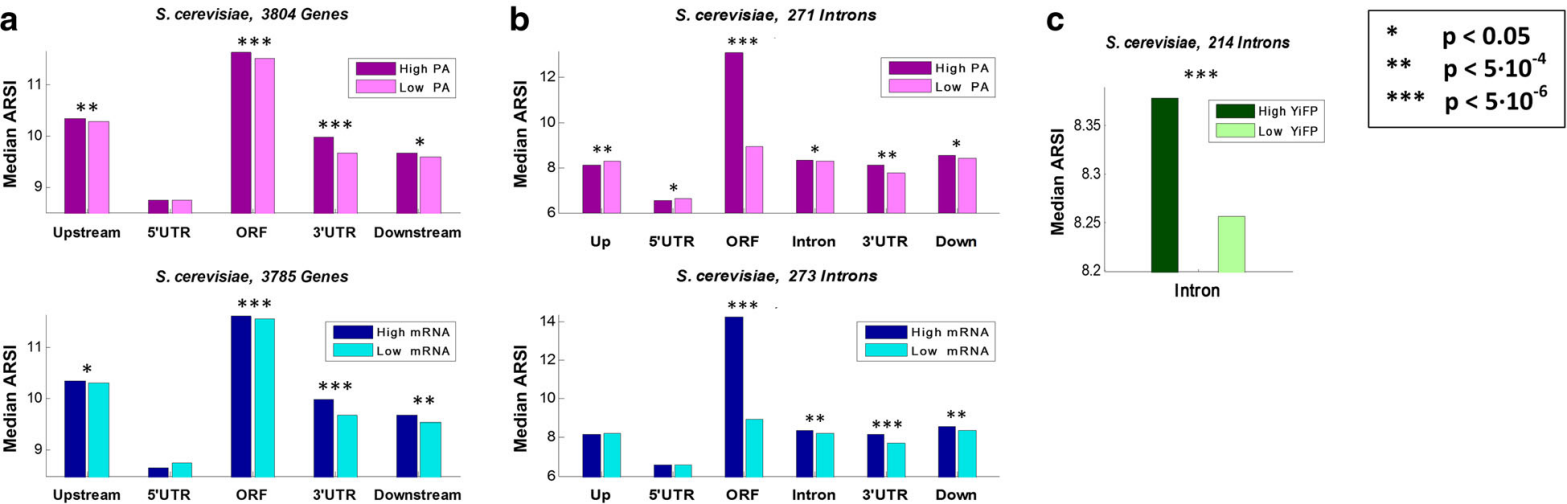
Analysis of the average *ARSI* measure for *S. cerevisiae*, while using various subgroups and for transcript regions. Highly expressed vs. lowly expressed genes, based on PA and mRNA levels (**a**; top and bottom, respectively); introns in highly expressed vs. lowly expressed genes, based on PA and mRNA levels (**b**; top and bottom, respectively), and YiFP measurement in the synthetic library (**c**; see Methods and [41]). Significant locations are presented in asterisks; see full details in Additional file 2: Table S3

Finally, we found that in both *E. coli* and *S. cerevisiae*, highly express genes tend to have higher *ARSI* scores in most of their genetic regions (Fig. 4; *p* < 0.05, Wilcoxon rank-sum test) including ORFs, introns, and 3’UTRs; see full details in Additional file 2: Table S3. Interestingly, this is also true when considering YiFP synthetic libraries (*p* = 5.17 · 10^−5^). This suggests that the *ARSI* score can be used for ranking genetic regions according to the expression levels of the genes they are encoded and/or their effect on expression levels based only on the genome in an unsupervised manner.

## Discussion

In this study we examine for the first time various regions in the gene that contain hidden information related to gene expression regulation, and especially to the transcription, splicing, and translation steps. Specifically, we report for the first time regions in the genome with elevated gene expression code concentration; these regions are expected to have significant regulatory effect on gene expression. Our analysis supports the conjecture that we are able to rank genetic elements according to their gene expression levels based on the *ARSI* score. This ranking is exclusively based on their sequence composition without any additional information, probably captures their ‘optimality’ in terms of fitting to the gene expression machinery, and can be implemented to better understand un-studied genomes.

Our analyses suggest that the *ARSI* (or an improved version of the *ARSI* approach) reported here can be used in genomic studies for various objectives. For example, it can be used for ranking genes, promoters, UTRs, and introns in organisms (including viruses and metagenomics data) with no gene expression measurements according to their potential expression levels, or ‘optimality’, based on the *ARSI* measure. This can promote inferring the function of the genes and encourage developing various systems biology models; in addition, it can be used for developing and engineering synthetic systems with improved gene expression levels. The *ARSI* may also be improved, e.g., via optimizing the weighting of different repetitive length and the number of times they appear in the genome.

It is important to emphasize that the reported *ARSI* measure correlation is only a first step towards further studying of the relation between *ARSI* (and more generally transcript nucleotide composition) and gene expression. This notion and other analyses done in this study (such as the analysis of *ARSI* for highly expressed vs. lowly expressed genes, comparison to randomized genome models, and Z-scoring), support our hypothesis that some of the examined regions include higher concentration of gene expression regulatory information; consequently, we were able to significantly rank genetic elements according to their ‘optimality’ based on the *ARSI* measure.

One way to better understand the strength/causality/directionality of the reported relations is via additional experimental analysis where regions with high *ARSI* levels are modified (e.g., using the emerging CRISPR/Cas9 technology) and the effect on gene expression is measured. Specifically, it will be interesting to understand the position-specific effect of some of the *ARSI* motifs on gene expression via the mentioned experiments. For example, it is possible that some splicing motifs could activate splicing when located downstream an exon, but repress splicing when located upstream of it. Our approach should be able to recognize these motifs if their sequence can be found in more than a single location in the reference genome, but would not indicate for any specific function, e.g. whether it is an enhancer or a repressor motif.

The *ARSI* approach can also be compared to regulatory motifs, identified via different experimental approaches; for example, it is expected to detect the most abundance motifs that are related to canonical expression regulation. On the other hand, it is possible that some known condition-specific motifs and splicing regulatory elements (SREs) would not be recovered in the *ARSI* screen; for example, motifs whose cognate factors are expressed at low levels in the cell may also be missed due to the focus on highly expressed or many genomic regions.

Finally, the results reported here suggest that various regions in the transcripts (including coding regions, UTRs, and introns) tend to include various gene expression codes. Thus, a related challenging topic for future research is the developing of molecular evolution models that incorporate those types of evolutionary constraints.

## Conclusions

Our analysis demonstrates that the *ARSI* unsupervised approach can be used for detecting and understanding gene expression codes in different parts of the genome/genes in previously un-studied organisms. These codes should be considered when developing novel models for genome and transcript evolution; they can be used for developing novel gene expression models and for gene expression engineering and synthetic biology systems.

## Additional files

Additional file 1: Figure S1. An illustration of various macro-molecules interacting with the mRNA transcript and regulatory signals interleaved in the genetic code. **Figure S2.** *ARSI* score distribution for the real and randomized models in various transcript regions for *S. pombe*. **Figure S3.** Generation scheme for *ARSI* measure using sliding windows of length *WL*. **Figure S4.** pre-mRNA exonic and intronic regions, basic definitions, and randomization models. **Figure S5.** Analysis of mRNA-seq and ribosomal profiling (Ribo-seq) measurements. Supplemental tables’ description. (PDF 1426 kb)
Additional file 2: Table S1.
*ARSI* correlation and statistics summary. **Table S2.** Meiotic cell cycle correlation summary. **Table S3.** Subgroups analysis. (XLSX 32 kb)

## Abbreviations

AA: Amino acid
ARSI: Average repetitive substring index
BS: Branch site
CDS: Coding sequence
CUB: Codon usage bias
NGS: Next generation sequencing
ORF: Open reading frame
PA: Protein abundance
RD: Ribosome density
SS: Splice site
TF: Transcription factor
UTR: UnTranslated region
YFP: Yellow fluorescent protein

## References

[1] Smale ST, Kadonaga JT (2003). The RNA Polymerase II Core Promoter. Annu Rev Biochem.

[2] Tuller T, Ruppin E, Kupiec M. Properties of untranslated regions of the *S. cerevisiae* genome. BMC genomics. 2009;10:391–1. 10.1186/1471-2164-10-391 PMC2737003 19698117

[3] Barash Y, Calarco JA, Gao W, Pan Q, Wang X, Shai O, Blencowe BJ, Frey BJ (2010). Deciphering the splicing code. Nature.

[4] Stergachis AB, Haugen E, Shafer A, Fu W, Vernot B, Reynolds A, Raubitschek A, Ziegler S, LeProust EM, Akey JM (2013). Exonic Transcription Factor Binding Directs Codon Choice and Affects Protein Evolution. Science.

[5] Alberts B, Johnson A, Lewis J, Morgan D, Raff M, Roberts K, Walter P: Molecular biology of the cell, Sixth edition edn: Garland Science; 2015

[6] Tuller T, Zur H (2015). Multiple roles of the coding sequence 5′ end in gene expression regulation. Nucleic Acids Res.

[7] Slesarev AI, Mezhevaya KV, Makarova KS, Polushin NN, Shcherbinina OV, Shakhova VV, Belova GI, Aravind L, Natale DA, Rogozin IB (2002). The complete genome of hyperthermophile *Methanopyrus kandleri* AV19 and monophyly of archaeal methanogens. Proc Natl Acad Sci.

[8] Su AAH, Tripp V, Randau L. RNA-Seq analyses reveal the order of tRNA processing events and the maturation of C/D box and CRISPR RNAs in the hyperthermophile *Methanopyrus kandleri*. Nucleic Acids Research. 2013;41(12):6250-6258. 10.1093/nar/gkt317 PMC3695527 23620296

[9] Dehal P, Satou Y, Campbell RK, Chapman J, Degnan B, De Tomaso A, Davidson B, Di Gregorio A, Gelpke M, Goodstein DM (2002). The Draft Genome of *Ciona intestinalis*: Insights into Chordate and Vertebrate Origins. Science.

[10] Suzuki MM, Nishikawa T, Bird A (2005). Genomic Approaches Reveal Unexpected Genetic Divergence Within *Ciona intestinalis*. J Mol Evol.

[11] Sasaki Y, Ishikawa J, Yamashita A, Oshima K, Kenri T, Furuya K, Yoshino C, Horino A, Shiba T, Sasaki T (2002). The complete genomic sequence of *Mycoplasma penetrans*, an intracellular bacterial pathogen in humans. Nucleic Acids Res.

[12] Ferrer-Navarro M, Gómez A, Yanes O, Planell R, Avilés FX, Piñol J, Pérez Pons JA, Querol E (2006). Proteome of the Bacterium *Mycoplasma penetrans*. J Proteome Res.

[13] Loftus BJ, Fung E, Roncaglia P, Rowley D, Amedeo P, Bruno D, Vamathevan J, Miranda M, Anderson IJ, Fraser JA (2005). The Genome of the *Basidiomycetous* Yeast and Human Pathogen *Cryptococcus neoformans*. Science.

[14] Janbon G, Ormerod KL, Paulet D, Byrnes EJ, Yadav V, Chatterjee G, Mullapudi N, Hon C-C, Billmyre RB, Brunel F (2014). Analysis of the Genome and Transcriptome of *Cryptococcus neoformans* var. *grubii* Reveals Complex RNA Expression and Microevolution Leading to Virulence Attenuation. PLoS Genet.

[15] Goordial J, Raymond-Bouchard I, Riley R, Ronholm J, Shapiro N, Woyke T, LaButti KM, Tice H, Amirebrahimi M, Grigoriev IV, Greer C, Bakermans C, Whyte L. Improved High-Quality Draft Genome Sequence of the *Eurypsychrophile Rhodotorula* sp. JG1b, Isolated from Permafrost in the Hyperarid Upper-Elevation McMurdo Dry Valleys, Antarctica. Genome Announcements. 2016;4(2). http://genomea.asm.org/content/4/2/e00069-16.full. 10.1128/genomeA.00069-16 PMC4796114 26988035

[16] Edgar R, Domrachev M, Lash AE (2002). Gene Expression Omnibus: NCBI gene expression and hybridization array data repository. Nucleic Acids Res.

[17] Katz Y, Wang ET, Airoldi EM, Burge CB (2010). Analysis and design of RNA sequencing experiments for identifying isoform regulation. Nat Meth.

[18] Chu Y, Corey DR (2012). RNA Sequencing: Platform Selection, Experimental Design, and Data Interpretation. Nucleic Acid Ther..

[19] Wang M, Weiss M, Simonovic M, Haertinger G, Schrimpf SP, Hengartner MO, von Mering C (2012). PaxDb, a Database of Protein Abundance Averages Across All Three Domains of Life. Mol Cell Proteomics.

[20] Ingolia NT, Ghaemmaghami S, Newman JRS, Weissman JS (2009). Genome-Wide Analysis in Vivo of Translation with Nucleotide Resolution Using Ribosome Profiling. Science.

[21] Johnson DS, Mortazavi A, Myers RM, Wold B (2007). Genome-Wide Mapping of in Vivo Protein-DNA Interactions. Science.

[22] Li N, Ye M, Li Y, Yan Z, Butcher LM, Sun J, Han X, Chen Q, Zhang X, Wang J (2010). Whole genome DNA methylation analysis based on high throughput sequencing technology. Methods.

[23] Hakim O, Misteli T. SnapShot: Chromosome Conformation Capture. Cell. 2012;148(5):1068–8. e1062. 10.1016/j.cell.2012.02.019 PMC6374129 22385969

[24] Diament A, Tuller T: Three-dimensional Genomic Organization of Genes’ Function in Eukaryotes. In: *Evolutionary Biology.* Springer International Publishing Switzerland; 2016

[25] Zur H, Tuller T. Exploiting hidden information interleaved in the redundancy of the genetic code without prior knowledge. Bioinformatics. 2014;31(8):1161-1168. 10.1093/bioinformatics/btu797 25433697

[26] Lee PS, Lee KH (2003). *Escherichia coli*—a model system that benefits from and contributes to the evolution of proteomics. Biotechnol Bioeng.

[27] Berbee ML, Taylor JW. Fungal Molecular Evolution: Gene Trees and Geologic Time. In: Systematics and Evolution. Edited by McLaughlin DJ, McLaughlin EG, Lemke PA. Berlin, Heidelberg: Springer Berlin Heidelberg; 2001: 229-245.

[28] Cherry JM, Adler C, Ball C, Chervitz SA, Dwight SS, Hester ET, Jia Y, Juvik G, Roe T, Schroeder M (1998). SGD: Saccharomyces Genome Database. Nucleic Acids Res.

[29] Spingola M, Grate L, Haussler D, Ares M (1999). Genome-wide bioinformatic and molecular analysis of introns in *Saccharomyces cerevisiae*. RNA.

[30] Wood V, Harris MA, McDowall MD, Rutherford K, Vaughan BW, Staines DM, Aslett M, Lock A, Bähler J, Kersey PJ (2012). PomBase: a comprehensive online resource for fission yeast. Nucleic Acids Res.

[31] Wood V, Gwilliam R, Rajandream MA, Lyne M, Lyne R, Stewart A, Sgouros J, Peat N, Hayles J, Baker S (2002). The genome sequence of *Schizosaccharomyces pombe*. Nature.

[32] Wang Y, Liu CL, Storey JD, Tibshirani RJ, Herschlag D, Brown PO (2002). Precision and functional specificity in mRNA decay. Proc Natl Acad Sci.

[33] Nagalakshmi U, Wang Z, Waern K, Shou C, Raha D, Gerstein M, Snyder M (2008). The Transcriptional Landscape of the Yeast Genome Defined by RNA Sequencing. Science.

[34] Lewis NE, Cho B-K, Knight EM, Palsson BO (2009). Gene Expression Profiling and the Use of Genome-Scale In Silico Models of *Escherichia coli* for Analysis: Providing Context for Content. J Bacteriol.

[35] Lackner DH, Beilharz TH, Marguerat S, Mata J, Watt S, Schubert F, Preiss T, Bähler J (2007). A Network of Multiple Regulatory Layers Shapes Gene Expression in Fission Yeast. Mol Cell.

[36] Zafrir Z, Tuller T (2015). Nucleotide sequence composition adjacent to intronic splice sites improves splicing efficiency via its effect on pre-mRNA local folding in fungi. RNA.

[37] Zafrir Z, Zur H, Tuller T. Selection for reduced translation costs at the intronic 5′ end in fungi. DNA Research. 2016;23(4):377-394. 10.1093/dnares/dsw019 PMC4991832 27260512

[38] Kendall MG, Stuart A (1973). The Advanced Theory of Statistics, vol. 2, 3rd edn.

[39] Linshiz G, Yehezkel TB, Kaplan S, Gronau I, Ravid S, Adar R, Shapiro E. Recursive construction of perfect DNA molecules from imperfect oligonucleotides. Molecular Systems Biology. 2008;4(1):n/a–a. 10.1038/msb.2008.26 PMC2424292 18463615

[40] Shabi U, Kaplan S, Linshiz G, BenYehezkel T, Buaron H, Mazor Y, Shapiro E (2010). Processing DNA molecules as text. Syst Synth Biol.

[41] Yofe I, Zafrir Z, Blau R, Schuldiner M, Tuller T, Shapiro E, Ben-Yehezkel T (2014). Accurate, Model-Based Tuning of Synthetic Gene Expression Using Introns in *S. cerevisiae*. PLoS Genet.

[42] Martin M. Cutadapt removes adapter sequences from high-throughput sequencing reads. EMBnetjournal: Next Generation Sequencing Data Analysis. 2011;17(1):10-12.

[43] Langmead B, Trapnell C, Pop M, Salzberg SL (2009). Ultrafast and memory-efficient alignment of short DNA sequences to the human genome. Genome Biol.

[44] Nilsen TW (2003). The spliceosome: the most complex macromolecular machine in the cell?. BioEssays.

[45] Rogozin I, Carmel L, Csuros M, Koonin E (2012). Origin and evolution of spliceosomal introns. Biol Direct.

[46] Kozak M (1986). Point mutations define a sequence flanking the AUG initiator codon that modulates translation by eukaryotic ribosomes. Cell.

[47] Zur H, Tuller T. Transcript features alone enable accurate prediction and understanding of gene expression in *S. cerevisiae*. BMC Bioinf. 2013;14 Suppl 15:S1–1. 10.1186/1471-2105-14-S15-S1 PMC3852043 24564391

[48] Ramakrishnan V (2002). Ribosome Structure and the Mechanism of Translation. Cell.

[49] Hogan DJ, Riordan DP, Gerber AP, Herschlag D, Brown PO (2008). Diverse RNA-Binding Proteins Interact with Functionally Related Sets of RNAs, Suggesting an Extensive Regulatory System. PLoS Biol.

[50] Forman JJ, Coller HA (2010). The code within the code: microRNAs target coding regions. Cell cycle.

[51] Bartel DP (2004). MicroRNAs: Genomics, Biogenesis, Mechanism, and Function. Cell.

[52] Cannarozzi G, Schraudolph NN, Faty M, von Rohr P, Friberg MT, Roth AC, Gonnet P, Gonnet G, Barral Y (2010). A Role for Codon Order in Translation Dynamics. Cell.

[53] Gu W, Zhou T, Wilke CO (2010). A Universal Trend of Reduced mRNA Stability near the Translation-Initiation Site in Prokaryotes and Eukaryotes. PLoS Comput Biol.

[54] Churchman LS, Weissman JS (2011). Nascent transcript sequencing visualizes transcription at nucleotide resolution. Nature.

[55] Li G-W, Oh E, Weissman JS (2012). The anti-Shine-Dalgarno sequence drives translational pausing and codon choice in bacteria. Nature.

[56] Zur H, Tuller T (2013). New Universal Rules of Eukaryotic Translation Initiation Fidelity. PLoS Comput Biol.

[57] Ghaemmaghami S, Huh W-K, Bower K, Howson RW, Belle A, Dephoure N, O'Shea EK, Weissman JS (2003). Global analysis of protein expression in yeast. Nature.

[58] Ares M, Grate L, Pauling MH (1999). A handful of intron-containing genes produces the lion's share of yeast mRNA. RNA.

[59] Brar GA, Yassour M, Friedman N, Regev A, Ingolia NT, Weissman JS (2012). High-Resolution View of the Yeast Meiotic Program Revealed by Ribosome Profiling. Science.

