# Supplementary material for: Unsupervised detection of regulatory gene expression information in different genomic regions enables gene expression ranking
Source: BMC Bioinformatics. 2017 Feb 1;18:77. doi: 10.1186/s12859-017-1497-z (PMC5286865; doi:10.1186/s12859-017-1497-z)
Supplement: Additional file 1: Figure S1. — An illustration of various macro-molecules interacting with the mRNA transcript and regulatory signals interleaved in the genetic code. Figure S2. ARSI score distribution for the real and randomized models in various transcript regions for S. pombe. Figure S3. Generation scheme for ARSI measure using sliding windows of length WL. Figure S4. pre-mRNA exonic and intronic regions, basic definitions, and randomization models. Figure S5. Analysis of mRNA-seq and ribosomal profiling (Ribo-seq) measurements. Supplemental tables’ description. (PDF 1426 kb) [file 12859_2017_1497_MOESM1_ESM.pdf]

Supplemental Figures

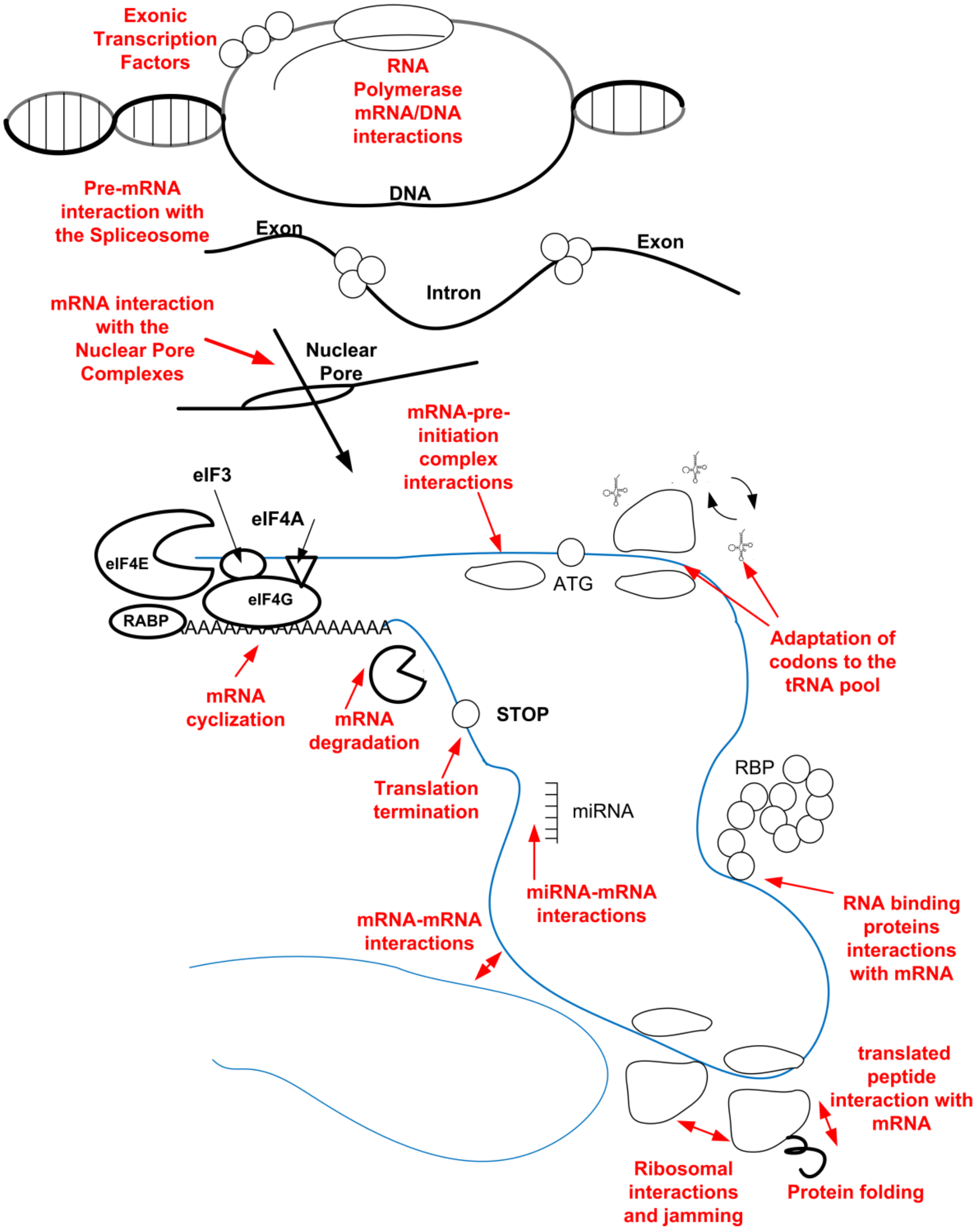

Figure S1: An illustration of various macro-molecules interacting with the mRNA transcript and regulatory signals interleaved in the genetic code.

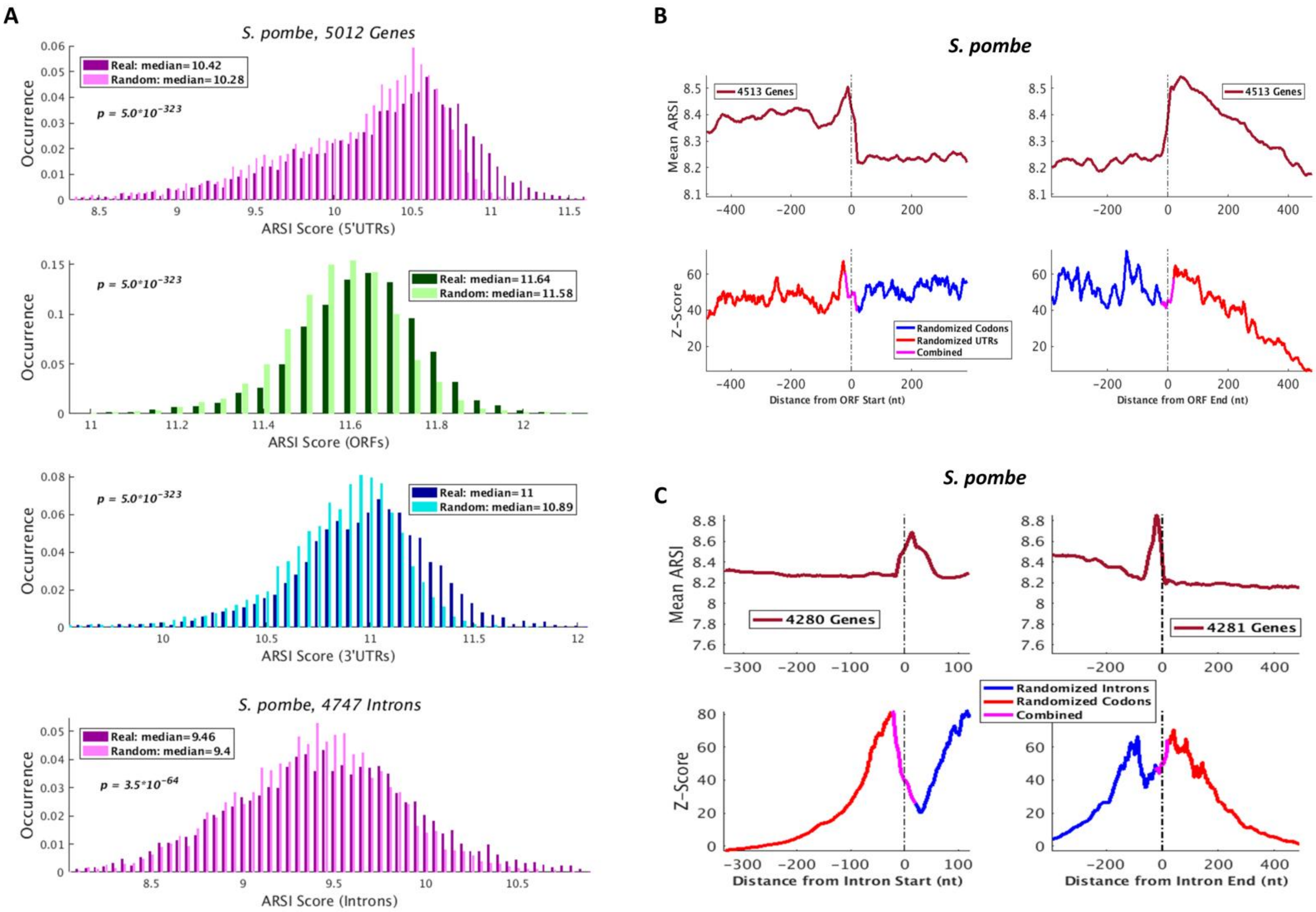

Figure S2: A) *ARSI* score distribution for the real and randomized models in various transcript regions for *S. pombe*. The *ARSI* values in the real transcriptome are significantly higher than the randomized models in all the examined regions (5'UTR/ORF/3'UTR/Intron; $p<3.5\cdot10^{-64}$, Wilcoxon signed-rank test). These results indicate that the real sequences tend to include longer substrings in comparison to the randomized ones. **B-C) Information concentration (*ARSI*) and selection (Z-score) profiles in various transcript regions *S. pombe*. The sequences are aligned to the ORF'S start, 5'SS, 3'SS, and ORF end.** The profiles for the mature mRNA (B) and for the pre-mRNA (C) show that more information is found in the ORF start, rather than downstream in the ORF; around the intronic splice sites the signal is stronger, as well as downstream from the ORF's end. In addition, the selective pressure on the transcript sequence is stronger in these locations. This suggests the possible enrichment of regulatory sequence motifs in these regions; the distance from the ORF/5'SS/3'SS is relative to the center of the sliding window; sliding window size is 41nt.

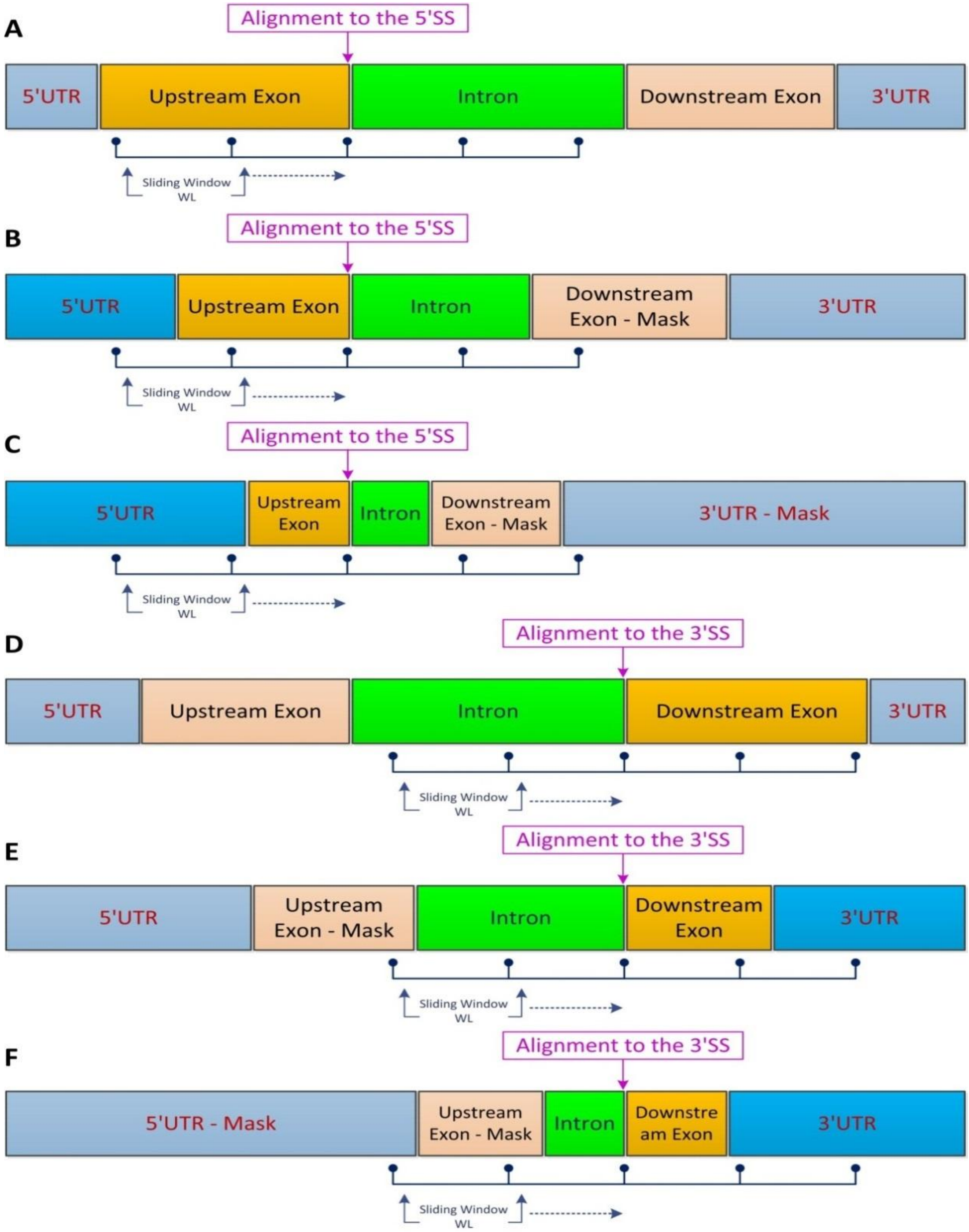

Figure S3: Generation scheme for *ARSI* measure using sliding windows of length WL, and showing some possible exon-intron boundary cases for an individual intron. Sequences are aligned around the donor site (A-C) and around the acceptor site (D-F). As illustrated, relevant UTR information is added, while downstream exons in 5'SS alignment and upstream exons in 3'SS alignment are masked. The information in various genomic elements is combined to form the mean profile. The following sliding windows sizes were used: 31nt, 41nt, 51nt, and 71nt, with a single nucleotide slide.

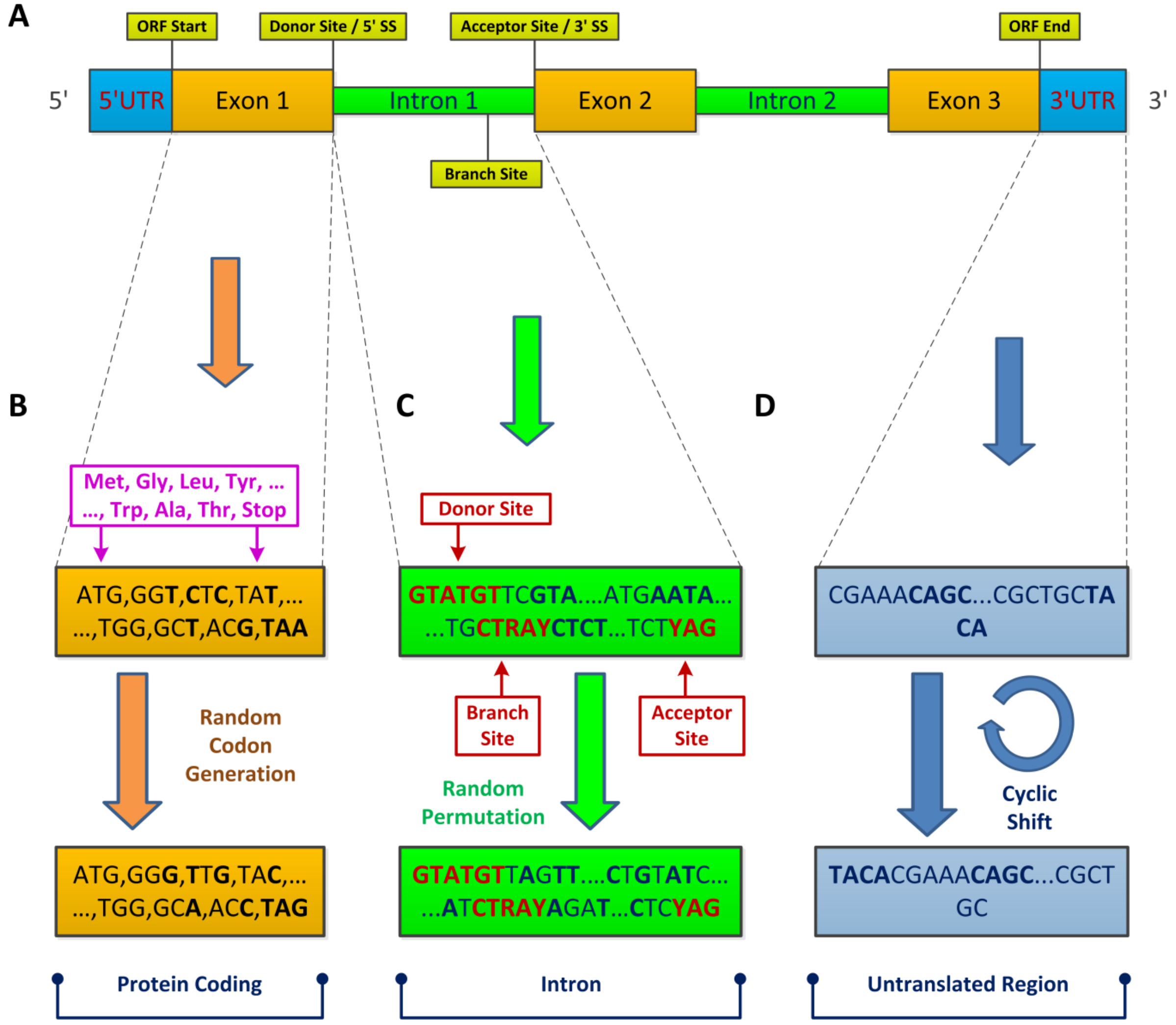

Figure S4: pre-mRNA exonic and intronic regions, basic definitions, and randomization models. A) The analyzed genetic elements can be divided in three major regions: untranslated regions (UTRs), exons, and introns (we did not consider UTR introns since in the analyzed organisms few introns appear in the UTRs; *e.g.* less than 6% in the case of *S. cerevisiae*). The introns include three canonical consensus sequences: the donor (or 5'SS; subsequence *GTAHGT*) and acceptor (or 3'SS; subsequence *YAG*) that define the intronic boundaries, and the branch site (BS; subsequence *CTRAY*) that is required for the lariat formation; those sequences are preserved in all our randomization models. B-D) In order to demonstrate that the reported features are in preference in endogenous transcripts, we compared the intronic sequences to the ones obtained by the following randomized models: B) encoded protein information is maintained; synonymous codons are generated based on their whole genome codon frequencies; C) uniform permutation of intronic nucleotides; D) UTRs randomization maintains GC content using cyclic shift; all the randomization models preserve the ATG context and intronic consensus sequences (5'SS/BS/3'SS), as well as additional exonic and intronic characteristics (see Methods for more details).

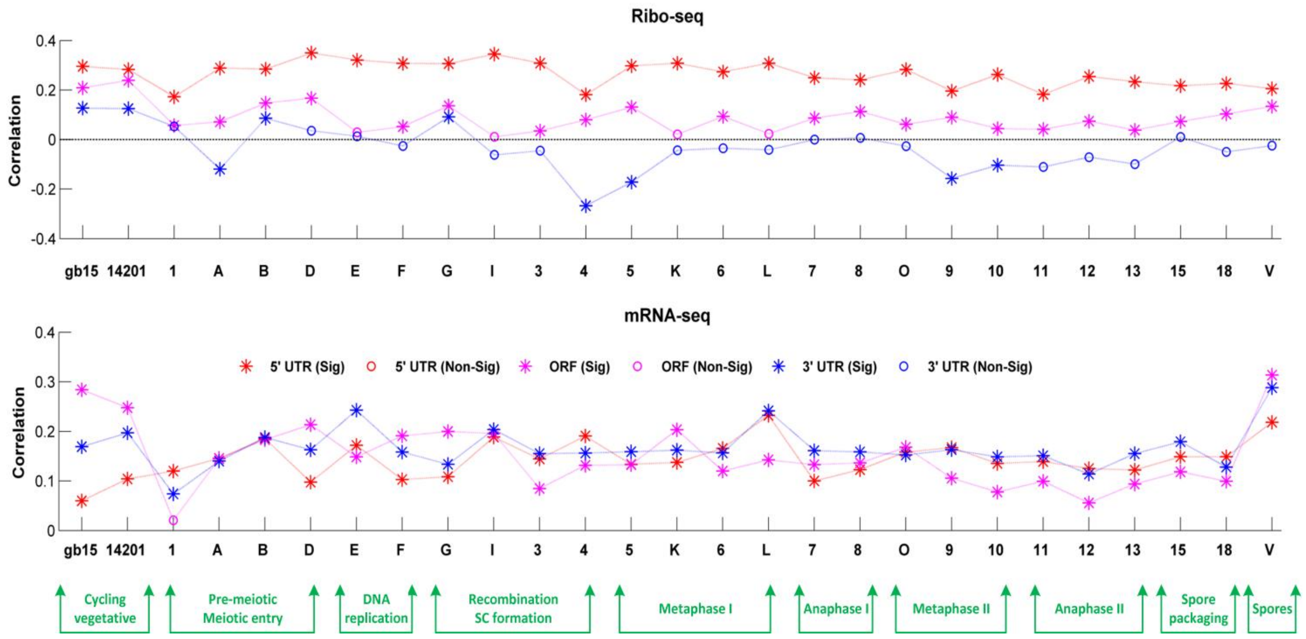

Figure S5: Analysis of mRNA-seq and ribosomal profiling (Ribo-seq) measurements. The correlation between the *ARSI* score and the RNA-seq / Ribo-seq data varies along the cell cycle with a correlation of up to 0.31/0.35 ($p<1.6\cdot10^{-6}$ and $p<3\cdot10^{-2}$, respectively); see also Supplementary **Table S2**. The significant time points with the highest correlation values are V/D and with the lowest correlation values are 12/4, respectively. However, the correlation usually seems relatively similar across the different conditions. This may suggests that, at least in this example, the gene expression information detected by the *ARSI* corresponds in a uniform manner to different meiotic cell cycle stages.

Supplemental tables' description

Table S1: ARSI correlation and statistics summary

This table summarizes the correlation results (Spearman) between the *ARSI* scores of the analyzed organisms (in various genomic regions), and gene expression measurements (based on PA, mRNA levels, and YiFP levels in a synthetic *S. cerevisiae* system; see Methods). It also includes the average and standard deviation of the *ARSI* scores for the actual and randomized models (in various genomic regions).

Table S2: Meiotic cell cycle correlation summary

This table summarizes the correlation results (Spearman) between the *ARSI* scores in the ORF region and UTRs, and the Ribo-seq / mRNA-seq measurements in various meiotic stages (Cycling vegetative, Pre-meiotic / Meiotic entry, DNA replication, Recombination / SC formation, Metaphase I, Anaphase I, Metaphase II, Anaphase II, Spore packaging, and Spores) and time points in *S. cerevisiae*, taken from the Brar et al. experiment; see Methods for details.

Table S3: Subgroups analysis

This table summarizes the median *ARSI* values of the analyzed organisms, for highly expressed *vs.* lowly expressed gene subgroups (based on PA, mRNA levels, and YiFP levels in a synthetic *S. cerevisiae* system). The P-values between the subgroups were calculated using Wilcoxon rank-sum test.
